# The positive psychology of AI: a systematic review of generative AI's impact on EFL learners' motivation, anxiety, and well-being in higher education

**DOI:** 10.3389/fpsyg.2026.1762411

**Published:** 2026-07-02

**Authors:** Yitong Dong, Kaiyu Zhang, Siou Li, Xiaoyu Gu

**Affiliations:** School of Foreign Languages, Changchun Institute of Technology, Changchun, China

**Keywords:** anxiety, EFL (English as a foreign language), emotions, Generative AI (GenAI), higher education, motivation, positive psychology

## Abstract

The integration of Generative AI (GenAI) into English as a Foreign Language (EFL) teaching is rapidly expanding, yet its effects on learners' affective states—key to language acquisition from a positive psychology lens—remain inadequately summarized. This systematic review synthesizes empirical evidence on how GenAI tools influence motivation, anxiety, and well-being among EFL learners in higher education. Following PRISMA guidelines, 1,420 records were identified from four databases (2019–2025), with 29 studies meeting inclusion criteria after screening. Results reveal a generally positive affective impact: GenAI enhances motivation and engagement, while typically reducing anxiety—though one study noted increased speaking anxiety. Benefits also extend to well-being, including emotional support and mindfulness. However, effects are moderated by factors such as gender (higher affective scores and AI self-efficacy in males) and academic level (lower perceived utility among Master's students). The findings affirm that well-designed GenAI aligns with Self-Determination Theory by supporting autonomy, competence, and relatedness. In conclusion, GenAI shows strong potential to foster positive psychological outcomes in EFL learning, yet its implementation must be intentionally tailored and inclusively designed to address contextual and individual differences.

## 1 Introduction

The integration of Generative Artificial Intelligence (GenAI) in higher education is triggering a profound paradigm shift, exerting a disruptive influence on the teaching practices of skill-oriented disciplines, particularly English as a Foreign Language (EFL). From adaptive learning platforms to intelligent conversational agents, GenAI tools have significantly expanded the boundaries of traditional education by offering personalized learning pathways and instant feedback. However, the cutting-edge of current research has shifted beyond the functional realization of the technology itself to a more central issue: how these intelligent technologies reshape learners' intrinsic psychological worlds and, consequently, influence ultimate educational outcomes. Understanding the psychological mechanisms underlying this “human-machine collaboration” process has become the key to propelling educational AI from being merely “usable” to truly “user-friendly.”

Moreover, it is important to distinguish “AI-assisted learning” from “GenAI learning”. AI-assisted learning is an umbrella term encompassing any use of AI (including rule-based, predictive, or generative) to support learning. In contrast, GenAI learning is a subset that specifically involves generative models (e.g., large language models, transformers) capable of producing novel, contextually responsive outputs, thereby enabling open-ended dialogue, personalized content creation, and real-time adaptive scaffolding. This review deliberately focuses on GenAI learning to maintain theoretical coherence.

In the realm of language learning, successful language acquisition is far from a mere accumulation of cognitive skills; it is deeply rooted in psychological factors such as learners' emotions and motivations (Dörnyei, 1998; Dewaele, 2011). Positive psychology theory emphasizes that positive psychological states, including motivation, anxiety, self-efficacy, and psychological well-being, are core determinants of whether learners can maintain long-term engagement and achieve success. Traditional EFL teaching environments often lack personalized support and authentic language use contexts, which can easily induce “language anxiety” in students and undermine their intrinsic motivation (MacIntyre, 2003; Coleman, 1997). Although a substantial body of research has explored the role of GenAI in enhancing language accuracy and fluency, there remains a lack of clear consensus in academia regarding how it systematically impacts these critical psychological constructs (Zhang and Liu, 2025; Griche and Bennis, 2026). This imbalance between cognitive and affective research limits our ability to comprehensively evaluate the educational efficacy of GenAI and hinders the full realization of its potential.

The purpose of this study is to conduct a systematic review of existing literature on the use of GenAI tools in EFL learning, specifically within the context of higher education. This analysis aims to identify the types of GenAI tools used, their affective mechanisms, implementation contexts, moderating factors, and psychological outcomes relevant to enhancing motivation, reducing anxiety, and promoting well-being among EFL learners. The significance of this study lies in constructing a comprehensive framework that connects technology (AI), theory (psychology), and practice (education). It not only seeks to validate the utility of GenAI as a teaching tool but also attempts to elucidate, from psychological perspectives, drawing on self-determination theory as the guiding framework, how it functions by fulfilling learners' fundamental psychological needs for autonomy, competence, and relatedness. The findings of this review will provide evidence-based guidance for educators, instructional designers, and policymakers to ensure that the implementation of GenAI is technologically advanced, psychologically supportive, and educationally effective, ultimately fostering an intelligent educational paradigm that is adaptive, learner-centered, and informed by psychological principles.

## 2 Method

### 2.1 Research design

This study employed a systematic review methodology following the Preferred Reporting Items for Systematic Reviews and Meta-Analyses (PRISMA) guidelines (Parums, 2021). The design was selected to comprehensively identify, critically appraise, and synthesize the existing empirical evidence on the influence of Generative AI on key educational psychology constructs. A meta-analysis was not feasible due to the heterogeneity in interventions, measurements, and outcome reporting among the included studies; therefore, a systematic narrative synthesis was conducted.

### 2.2 Inclusion and exclusion criteria

The study selection was governed by a predefined PICOS framework to ensure focus and relevance.

(1) Population: Included learners in formal or informal educational settings. Excluded studies focusing solely on participants in teacher training. (2) Intervention: Included the use of Generative AI (e.g., ChatGPT, advanced AI chatbots) or conversationally adaptive AI tools. Excluded studies using only rule-based or simple adaptive tutoring systems without generative capabilities. (3) Comparator: Not required for inclusion. (4) Outcomes: Included studies must have reported measurable outcomes related to motivation (e.g., intrinsic motivation, self-efficacy, anxiety), cognition/metacognition (e.g., strategy use, feedback processing), or beliefs about learning (e.g., self-efficacy, perceptions of AI). Studies reporting only on linguistic proficiency or academic achievement without linking to these psychological constructs were excluded. (5) Study Design: Included empirical studies (quantitative, qualitative, mixed-methods). Excluded theoretical papers, literature reviews, and commentaries.

### 2.3 Data collection method

**Figure 1:**
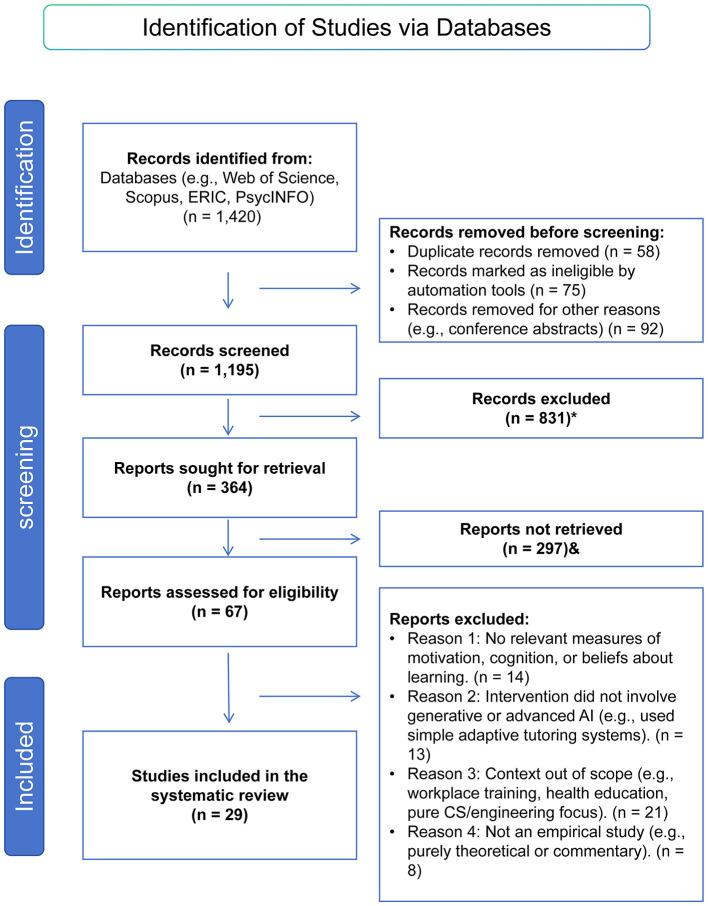
The workflow of the selection. *Out of scope (e.g., focused solely on vocational training, language acquisition outcomes, or higher education administration without addressing core educational psychology constructs). ^&^Reasons: primarily due to inability to access the full text through institutional subscriptions or inter-library loan, or because the publication was not in English.

The data collection process involved a systematic literature search and a multi-stage screening and extraction protocol. The work flow is showed in Figure 1.

#### 2.3.1 Identification process

An initial systematic search was conducted across four major scholarly databases (Web of Science, Scopus, ERIC, and PsycINFO). The search strategy was meticulously designed using a combination of free-text keywords and controlled vocabulary to capture the intersection of three core concepts: (1) Generative AI Technologies (e.g., “Generative AI”, “ChatGPT”, “large language model”), (2) Educational Context (e.g., “education”, “learning”, “higher education”), and (3) Psychological Constructs (e.g., “motivation”, “anxiety”, “self-efficacy”, “well-being”, “cognition”). These terms were combined with Boolean operators (AND/OR), and the search was limited to the period from January 2019 to November 2025, ultimately yielding 1,420 initial records.

#### 2.3.2 Screening process

All identified records from databases (*n* = 1,420) were imported into a reference management software (Endnote 2025). A total of 225 records were removed prior to screening, including 58 duplicate records, 75 records marked as ineligible by automation tools, and 92 records removed for other reasons (e.g., conference abstracts). The titles and abstracts of the remaining 1,195 records were screened by two independent reviewers against the inclusion criteria. This stage primarily focused on excluding works that were clearly out of scope based on population, intervention, or outcome, including non-generative AI studies which were intentionally excluded, resulting in the exclusion of 831 records. The primary reason for exclusion at this stage was a focus on contexts outside the scope of core educational psychology (e.g., technical AI development).

#### 2.3.3 Eligibility assessment

The full texts of 364 potentially relevant articles were retrieved. Of these, 298 reports were excluded as the full text was unavailable or not in English. The remaining 67 reports underwent a rigorous, full-text eligibility assessment by two independent reviewers. The assessment applied a two-tiered exclusion logic: (1) Conceptual Misalignment: Studies were excluded if they did not measure core educational psychology constructs (e.g., motivation, anxiety, well-being, self-efficacy) but instead reported only usability, satisfaction, or system performance metrics (*n* = 14). Also excluded were studies conducted in out-of-scope contexts, such as vocational training without an English as a Foreign Language (EFL) focus, or higher education administration studies that did not assess learner psychological outcomes (*n* = 21). (2) Methodological Misalignment: Studies were excluded if they did not involve a genuine generative AI intervention (defined as AI systems based on large language models or transformer architectures capable of generating novel, human-like text, dialogue, or personalized content). Examples of exclusion included studies using only hypothetical scenarios, non-interactive AI descriptions, or pre-GenAI rule-based systems without generative capabilities (*n* = 13). Additionally, non-empirical works such as theoretical papers, opinion pieces, commentaries, or conference abstracts without original data were excluded (*n* = 8). (3) This process culminated in the final inclusion of 29 studies.

#### 2.3.4 Data extraction

A standardized extraction form was developed a priori, containing: (1) author(s) and year, (2) AI tool/platform, (3) target skill(s), (4) context and sample size, (5) quantitative results (means, p-values, effect sizes if reported), and (6) affective/learning outcome. Two independent reviewers extracted data from each of the 29 studies. Discrepancies were resolved by consensus or by consulting a third reviewer. For studies that reported only qualitative findings or did not provide extractable numerical data (marked as “N/A” in Table 1), the narrative findings were described in the synthesis, and no numerical imputation was performed.

**Table 1:** Studies on GenAI's affective and skill impact on EFL learners in higher education.

No	Author(s) and year	AI tool/platform	Target skill(s)	Context/sample size (*N*)	Quantitative results (numerical data)	Affective/learning outcome
a. Conversational AI and chatbots (12 studies)						
1	Kohnke and Moorhouse (2025)	ChatGPT, Nearpod, Perplexity, Mizou, MagicSchool.ai	Motivation, emotions, well-being	First-year university students (*N =* 21) (7 F, 14 M)	N/A (qualitative thematic analysis)	Enhanced motivation; reduced anxiety/stress; fostered emotional support
2	Alfaleh et al. (2025)	AI-based tools, ChatGPT (Interaction)	Motivation, engagement, oral reading	University-level EFL learners (Saudi Arabia, UG/PG) (*N =* 297)	Study level difference *p =* 0.001. Motivation-AI correlation *r =* 0.623.	Master's students showed significantly lower positive perception than undergraduates
3	Dandu et al. (2024)	ChatGPT, Duolingo, Grammarly	Intrinsic motivation, autonomy, competence	B. Tech engineering students (India) (*N =* 120) (EG = 60, CG = 60)	Intrinsic motivation: EG 4.3 vs. CG 3.5 (*p < * 0.001). Autonomy: EG 4.1 vs. CG 3.4.	Significantly enhanced intrinsic motivation, autonomy, and competence
4	Al-Abdullatif et al. (2023)	Bashayer (Chatbot embedded in WhatsApp)	Motivation, self-efficacy	Postgraduate EFL learners (Saudi HE) (*N =* 60)	Total motivation mean: EG 4.03 vs. CG 3.34. Self-efficacy EG 3.99 vs. CG 3.37.	Positive impact on task value, self-efficacy, and overall motivation
5	Song and Song (2023)	ChatGPT	Academic writing skills, motivation	EFL students (Bachelor's Program, China) (*N =* 50)	N/A (mixed-methods study)	Enhanced academic writing skills and learning motivation
6	Wang and Xue (2024)	AI-Driven Chatbots (TalkAI, SpeakG, Wenxin Yiyan, Xunfei Xinghuo)	Academic engagement	EFL students (China) (N/A)	N/A (intervention study reporting significant positive effect)	Significantly enhanced EFL students' academic engagement
7	Yin et al. (2024)	Educational Chatbot	Intrinsic motivation, induced emotions	EFL students (N/A)	N/A (reported positive impact)	Emotion positively and significantly correlated with intrinsic motivation
8	Yuan and Liu (2025)	AI Tools (Various)	Engagement, enjoyment, motivation	EFL learners (N/A)	N/A (intervention study reporting significant positive effect)	Significantly enhanced EFL learners' engagement, enjoyment, and motivation
9	Wang and Wang (2024)	Large language models	Engagement	Chinese EFL learners (N/A)	N/A (Self-determination theory (SDT) perspective)	Examined engagement with LLMs from an SDT perspective
10	Prégent et al. (2025)	ChatGPT, NLP, ML models, AI chatbots, generative AI	Therapeutic training, content creation	*N =* 10	N/A (scoping review; no primary data)	Increased learner motivation and reduced instructional anxiety, while raising concerns about AI ethics that may impact educator well-being
11	Yilmaz and Yilmaz (2023)	ChatGPT (GPT-3.5)	Motivation	Undergraduate CS students (Turkey); *N =* 45 (EG = 21, CG = 24)	CT: EG 126.73 vs. CG 112.61 (*p =* 0.000, f = 0.309); Self-efficacy: EG 41.32 vs. CG 33.52 (*p =* 0.000, f = 0.265); Motivation: EG 96.50 vs. CG 85.17 (*p =* 0.002, f = 0.214)	Significantly enhanced motivation; improved creativity, algorithmic thinking, and problem-solving
12	Hawanti and Zubaydulloevna (2023)	ChatGPT	Writing skills, anxiety	Indonesian EFL university students; *N =* 73 (EG = 36, CG = 37)	Post-test anxiety: EG 2.895 vs. CG 3.100 (F = 4.137, ^*^p^*^= 0.030, η^2^ = 0.576)	Alleviating students' anxiety in english writing classroom
b. AI for speaking, listening, and writing skills (7 studies)						
1	Shazly (2021)	AI (implied ASR/Chatbot)	English speaking anxiety, performance	EFL learners (Case study) (N/A)	Mean anxiety score increased: pre 15.3, post 16.8	Negative outcome: learner anxiety score increased after AI use
2	Yang et al. (2024)	Pigai	Engagement with feedback, revision	EFL Students (China) (*N =* 5) (sophomores)	Error corrective feedback take-up rate: 49%. suggestions/tips take-up rate: 15%	Sustained engagement with AWE feedback facilitates improvement and potentially autonomy
3	Zou et al. (2023)	AI speaking apps (WeChat/Tencent Docs)	Speaking, interactional competence	EFL students (China) (*N =* 218)	PI_average: EG M = 3.92 vs. CG M = 2.35 (T = 8.63, *p =* 0.00)	Interaction activities in AI environment enhanced perception of Interaction
4	Zou et al. (2023a)	AI Speech Evaluation Program (ASR)	Speaking, acceptance	EFL Students (China) (*N =* 40)	N/A	Students reported acceptance of ASR for speaking practice
5	Zou et al. (2023b)	AI speaking apps (WeChat/Tencent Docs)	Speaking practice, willingness to communicate	EFL students (China)(*N =* 70)	88% punched cards; 80% agreed punching cards motivated practice	AI-assisted social network interaction supported speaking practice and motivation
6	Fathi and Rahimi (2024)	AI-enhanced writing mediation (GenAI)	Academic writing skills, perceptions	EFL learners (IELTS preparation) (N/A)	N/A (qualitative study)	Improved writing skills; generated positive perceptions
7	Xiao (2025)	AI-driven Speech Recognition (ASR)	Listening, anxiety, flow experience	EFL learners (Hainan University) (*N =* 84)	N/A (RCT study reporting significant impact)	Significant impact on anxiety and flow experience (positive affective influence reported)
c. Adaptive platforms, surveys, reviews, and other AI tools (10 studies)						
1	Shu and Xu (2022)	Tencent Translator, Baidu AI Platform (AI devices)	Autonomous learning, adaptability	Higher education (university students, China) (*N =* 552)	Average adaptability: 87.83% (MOTH-FLAME model). 4th year fitness 3.44.	Cultivated stable benign learning motivation; increased interest
2	Khasawneh et al. (2025)	AI-assisted portfolio assessment	AER, mindfulness, attitude	EFL students (Bahir Dar University, Ethiopia) (*N =* 69)	AER *p =* 0.00 (F = 19.26). Attitude mean score: 4.02 (5-point scale)	Significant enhancement of AER and mindfulness; highly positive attitude
3	Huang and Wang (2023)	AI Agent (personalized recommendation)	Engagement, motivation, achievement	EFL learners (*N =* 102) (EG = 43, CG = 59)	N/A	Significantly enhanced learning engagement for moderately motivated students
4	Puzon et al. (2025)	General AI system	Affective component, AI self-efficacy	Engineering students (Philippines) (*N =* 254)	Affective component (gender diff): male M 3.44, female M 3.22 (*p =* 0.029). AI self-efficacy *p =* 0.002.	Males scored significantly higher than females in affective component and AI Self-Efficacy
5	Batdi and Jibril (2024)	AI applications (general)	Academic achievement	HE (Meta-analysis, K = 5 studies)	Effect size (hedges' g): g = 0.63. heterogeneity: I^2^ = 94.31	AI applications had a medium effect on learning achievement
6	Shi et al. (2025)	Translation software, ChatGPT, vocabulary apps	User preference, affective needs	EFL students (China) (*N =* 269) (53.2% F, 46.8% M)	Translation software usage 67.8%. demand for personalized learning paths 79.1%	Strong preference for utility tools. High demand for personalized and automated feedback systems
7	Wei (2023)	AI-mediated instruction (AI platform)	L2 motivation, self-regulated learning (SRL)	Undergraduates (China) (N/A)	L2 motivation: group × time *p =* 0.04 (η^2^ = 0.11). SRL: group × time *p =* 0.00 (η^2^ = 0.38)	Positive influence on L2 motivation and SRL (significant improvements)
8	Weng and Chiu (2023)	Intelligent CALL (ICALL)	Instructional design, learning outcomes	EFL students (N/A)	N/A	Identified gaps in ICALL environment design related to personalization and affective states
9	Zawacki-Richter et al. (2019)	General AI (pre-GenAI)	Assessment, adaptive systems	Higher education (systematic review 2007–2018) (*N =* 146)	N/A	Identified assessment and personalization as key AI roles; noted the importance of affective factors
10	Dou and Sun (2025)	AI tools (general)	Motivation, psychological well-being (PWB), psychological capital (PsyCap)	EFL students (China) (N/A)	ANCOVA results: experimental group showed significantly higher post-test scores in motivation, PsyCap, and PWB compared to the control group (*p < * 0.05).	Enhanced students' motivation, psychological well-being, and psychological capital

AER, Academic Emotion Regulation; CT, Computational Thinking; EG, Experimental Group; CG, Control Group.

#### 2.3.5 Synthesis approach

A narrative synthesis was conducted because meta-analysis was precluded by heterogeneity in interventions (ChatGPT, Pigai, Bashayer chatbot, ASR systems, etc.), outcome measures (different motivation scales, anxiety inventories, well-being indices), and study designs (RCTs, quasi-experimental, qualitative, mixed-methods). We followed the Synthesis Without Meta-analysis (SWiM) reporting guidelines. Findings were organized thematically around three core affective constructs: motivation, anxiety, and well-being. Within each theme, we further structured the synthesis by moderating factors (gender, academic level) and by AI functional features (personalized recommendation, automated feedback, conversational interaction).

#### 2.3.6 Quality appraisal

The methodological quality of the 29 included studies was critically appraised by two independent reviewers. Depending on the study design, the appropriate checklist (e.g., for RCTs, quasi-experimental studies, or qualitative research) was employed. The appraisal focused on key aspects such as sampling strategy, measurement validity, control for confounding, and appropriateness of analytical methods. To assess inter-rater reliability, we calculated Cohen's kappa (κ) for the overall risk-of-bias classification (low / moderate / high) of the 25 empirical studies (four systematic reviews / theoretical papers were not rated). The observed agreement rate was 92% (23 out of 25 studies rated identically), and the expected chance agreement (P_e_) was 0.47, yielding κ = 0.84, which indicates “almost perfect” agreement beyond chance.

Disagreements were resolved through discussion, when consensus could not be reached (fewer than 5% of cases), a third reviewer (the corresponding author) made the final decision. The results of the quality appraisal were used not to exclude studies, but to inform the interpretation of findings and assess the overall strength and confidence of the synthesized evidence. Studies were categorized as having low, moderate, or high risk of bias. A complete MMAT (Mixed Methods Appraisal Tool, version 2018) scoring table for all 29 studies, including individual criterion ratings and overall risk of bias, is provided in Appendix A and was used to qualify the strength of each finding (e.g., higher confidence for low-bias studies; tentative conclusions for moderate-/high-bias studies).

## 3 Results

### 3.1 Summary of included studies

The 29 included studies encompassed a diverse range of AI tools, including conversational agents like ChatGPT and specialized chatbots (e.g., Bashayer on WhatsApp), Automated Writing Evaluation systems (e.g., Pigai), AI-powered speech apps (e.g., on WeChat/Tencent Docs), and comprehensive AI platforms. Sample sizes varied widely, from small-scale case studies (*N* = 5) to larger surveys (*N* = 552). The studies were conducted in various Asian and Middle Eastern EFL contexts, including China, Saudi Arabia, India, Ethiopia, and the Philippines. The outcomes were primarily measured through pre-post tests, surveys, and qualitative interviews. Details are showed in Table 1.

### 3.2 Synthesis of findings on affective constructs

#### 3.2.1 Impact on motivation and engagement

The synthesis demonstrates robust evidence indicating the positive impact of GenAI tools on learner motivation and engagement, in accordance with the tenets of positive psychology. Quantitatively, Dandu et al. (2024) observed significantly elevated intrinsic motivation in the experimental group utilizing AI tools (M = 4.3) in contrast to the control group (M = 3.5) (*p* < 0.001), with this beneficial effect also extending to measures of autonomy and competence Dandu et al., (2024). Likewise, Al-Abdullatif et al. (2023) reported that the Bashayer chatbot significantly enhanced overall motivation (experimental group M = 4.03 vs. control group M = 3.34) and self-efficacy (experimental group M = 3.99 vs. control group M = 3.37), providing further evidence of AI-facilitated positive affective outcomes (Al-Abdullatif et al., 2023). Additionally, Yilmaz and Yilmaz (2023) provided strong evidence that ChatGPT (GPT-3.5) significantly enhanced learner motivation (*p* = 0.002) and self-efficacy (*p* = 0.000), while simultaneously improving high-order cognitive skills such as algorithmic thinking and problem-solving among undergraduate students (Yilmaz and Yilmaz, 2023). Moreover, Wang and Xue (2024) and Yuan and Liu (2025) reported that AI-driven chatbots and tools significantly enhanced academic engagement, enjoyment, and motivation among EFL learners (Wang and Xue, 2024; Yuan and Liu, 2025). In writing-intensive contexts, GenAI mediation demonstrated a dual benefit for both affect and proficiency. Song and Song (2023) utilized a mixed-methods design to show that ChatGPT effectively enhanced both academic writing skills and learning motivation (Song and Song, 2023). This is corroborated by the qualitative evidence from Fathi and Rahimi (2024), who found that AI-enhanced writing mediation (GenAI) not only improved technical writing skills but also generated consistently positive perceptions among IELTS students (Fathi and Rahimi, 2024).

#### 3.2.2 Impact on anxiety

The evidence concerning anxiety presents a more intricate pattern that cannot be reduced to a simple “reduces” or “increases” conclusion. Critically, the effect of GenAI on anxiety depends on specific design and contextual factors, not on the technology itself. For most learners, AI tools reduce anxiety by creating a low-stakes, private practice environment. For example, Kohnke and Moorhouse (2025) found that GenAI reduced speaking anxiety by offering non-judgmental conversational partners (Kohnke and Moorhouse, 2025). Similarly, Zou et al. (2023) demonstrated that ASR-based speaking practice alleviates fear of negative evaluation, a primary source of language anxiety (Zou et al., 2023,a,b). In the context of writing instruction, Hawanti and Zubaydulloevna (2023) demonstrated that integrating ChatGPT as a learning partner effectively alleviated students' anxiety in the EFL writing classroom (*p* = 0.030,η ^2^ = 0.576), reinforcing its role as a psychologically supportive tool (Hawanti and Zubaydulloevna, 2023). Mechanistically, these beneficial effects operate through the satisfaction of the Self-Determination Theory (SDT) need for competence: learners can practice repeatedly without public scrutiny, gradually building mastery and self-efficacy.

However, the exception reported by Shazly (2021)—where speaking anxiety increased from 15.3 to 16.8 after AI intervention (Shazly, 2021)-requires critical explanation. We interpret this finding through the lens of technostress and negative cognitive appraisal. When an AI interface is perceived as inflexible, the tasks as overly demanding, or the interaction as high-pressure (e.g., real-time evaluation without sufficient scaffolding), learners may appraise the technology as a threat to their competence rather than a facilitator. This appraisal triggers anxiety instead of reducing it. Thus, the same AI tool can either reduce or increase anxiety depending on whether it is designed as a supportive scaffold (low stakes, private, formative) or as an assessment overseer (high stakes, public, summative).

In conclusion, while the weight of evidence leans toward an anxiety-reducing effect, this is not a universal property of AI. The critical moderator is human-computer interaction design: AI features that enhance perceived control, provide gradual difficulty, and avoid performance pressure are likely to reduce anxiety; those that do not may backfire. This nuanced understanding moves beyond simple description and offers actionable guidance for educators and designers.

#### 3.2.3 Impact on well-being and broader affective states

A relatively small yet noteworthy subset of studies has highlighted positive impacts on overall well-being and specific affective characteristics. In terms of emotional support and well-being, Kohnke and Moorhouse (2025) clearly established, through their qualitative analysis, a connection between AI use and the promotion of emotional support and well-being (Kohnke and Moorhouse, 2025). Regarding mindfulness and attitude, Khasawneh et al. (2025) discovered that AI—assisted portfolio assessment significantly improved Academic Emotion Regulation (AER) and mindfulness, with students exhibiting a highly positive attitude, as evidenced by a mean score of 4.02 out of 5 (Khasawneh et al., 2025). Dou and Sun (2025) showed comprehensive gains in Psychological Capital (PsyCap) and general well-being (Dou and Sun, 2025). As for flow and enjoyment, the induction of positive emotions and flow experiences, as reported by Xiao (2025) and Yin et al. (2024), further enriched the positive learning experience, thereby supporting psychological well-being (Yin et al., 2024; Xiao, 2025).

### 3.3 The moderating role of learner and tool characteristics

The review has indicated that the affective influence of AI is non—uniform and is subject to moderation by multiple factors. In terms of gender, Puzon et al. (2025) identified a significant gender disparity. Male engineering students scored higher than their female counterparts on the affective component (Male M = 3.44, Female M = 3.22) and in AI self—efficacy (Puzon et al., 2025). This implies that gender—specific support and training might be essential to ensure equitable benefits. Regarding academic level, Alfaleh et al. (2025) discovered that Master's students had a significantly less positive perception of the motivational utility of AI compared to undergraduate students (Alfaleh et al., 2025). This finding suggests that learner maturity and specific academic requirements affect the perceived value of AI. Concerning the type of AI tool, its utility and impact differed depending on the tool. Translation software and vocabulary apps were the most frequently used (Shi et al., 2025), whereas tools facilitating social interaction (Zou et al., 2023) were especially effective in enhancing motivation and the willingness to communicate (Zou et al., 2023,a).

Additionally, ethical perceptions varied, with concerns being particularly high about the use of ChatGPT in high—stakes exams (Sajawal and Kittur, 2024b,a). Prégent et al. (2025) noted that ethical concerns also contributed to instructional anxiety (Prégent et al., 2025). Specifically, the potential for plagiarism and the misrepresentation of student skills can transform the perception of AI from a supportive scaffold into a source of “integrity-related stress”. Beyond ethics, other studies reported additional findings. Zou et al. (2023) noted that AI tools can have a medium effect on learning achievement, while Batdi and Jibril (2024), Weng and Chiu (2023), and Zawacki-Richter et al. (2019) pointed out that many AI systems still lack adequate design features to address learners' emotional states (Batdi and Jibril, 2024; Zou et al., 2023; Weng and Chiu, 2023; Zawacki-Richter et al., 2019).

## 4 Discussion

This systematic review presents a comprehensive and nuanced exploration of the impact of Generative AI on language learning psychology—specifically the affective landscape of EFL learning in higher education. The results not only confirm the significant potential of Generative AI but also reveal a complex interplay of factors that shape its effectiveness, offering fresh perspectives on how to maximize its benefits while mitigating potential drawbacks.

### 4.1 Functional mapping of AI features to self-determination theory

The findings strongly support that well-designed GenAI tools align with the core principles of positive psychology and with Self-Determination Theory (SDT). SDT is a well-validated macro-theory of human motivation that centers on three universal psychological needs: autonomy (the experience of volition and psychological freedom), competence (the sense of mastery and effectiveness), and relatedness (the feeling of connection with others) (Ryan and Deci, 2024). For example, Wang and Wang (2024) examined this engagement through the lens of SDT, suggesting that LLMs foster active participation by satisfying learners' internal psychological needs (Wang and Wang, 2024). According to SDT, when social environments (including AI tools) support these three needs, intrinsic motivation and psychological well-being are enhanced; when the needs are thwarted, motivation declines and negative affect (e.g., anxiety, disengagement) increases (Ryan and Deci, 2024; Deci and Ryan, 2012). However, rather than merely claiming alignment, we specify how distinct AI features differentially satisfy the three basic psychological needs—autonomy, competence, and relatedness. The information is summarized in the Table 2 and the details see below:

- Autonomy through Personalized Learning Paths: Tools like ChatGPT empower learners by offering personalized learning paths, a feature demanded by 79.1% of university students (Shi et al., 2025). This shift from “external regulation” to “self-regulated learning” (SRL) fulfills the need for autonomy by allowing students to control the pace, difficulty, and content of their learning (Shu and Xu, 2022; Wei, 2023). Two studies provide clear, quantifiable evidence of autonomy-related outcomes: Shu and Xu (2022) directly measured learner demand for personalization (a proxy for autonomy preference) (Shu and Xu, 2022), while Wei (2023) empirically demonstrated the effect of AI on SRL (with a high effect size of η^2^ = 0.38)—a key behavioral manifestation of autonomy (Wei, 2023). Together, they illustrate how specific AI features (personalized learning paths and AI-mediated instruction) support the satisfaction of the autonomy need.
- Competence through Automated Feedback Mechanisms: AI-programmed Automated Writing Evaluation (AWE) and Speech Recognition (ASR) systems provide immediate, non-judgmental, and formative feedback. This mechanism allows for low-stakes iterative mastery, fulfilling the need for competence by enabling learners to cross their Zone of Proximal Development (ZPD) (the range of tasks a learner cannot yet do alone but can accomplish with appropriate support) rapidly (Van Compernolle and Williams, 2012; Fletcher, 2024). Quantitatively, the significantly higher intrinsic motivation (M = 4.3) and competence scores (M = 4.2) found in experimental groups demonstrate that immediate achievement cues directly reinforce the learner's sense of efficacy (Yang et al., 2024; Zou et al., 2023; Fathi and Rahimi, 2024).
- Relatedness through Conversational Interaction: Anthropomorphic chatbots and AI assistants that provide emotional cues and interactive negotiation mimic human social dynamics. By fostering a sense of “human-machine connection,” these tools reduce the isolation of independent practice, thereby satisfying the need for relatedness within a virtual learning community (Kohnke and Moorhouse, 2025; Al-Abdullatif et al., 2023; Zou et al., 2023).

**Table 2:** Summary of GenAI features to SDT psychological needs.

AI functional feature	Example tools (from Table 1)	Primary SDT need	Empirical support
Personalized recommendation and learner pacing	ChatGPT, Shu and Xu (2022); Wei (2023)	Autonomy	79.1% demand for personalized paths; SRL effect size η^2^ = 0.38
Immediate formative feedback and scaffolded difficulty	Pigai Yang et al. (2024); ASR Zou et al. (2023); Fathi and Rahimi (2024)	Competence	Intrinsic motivation EG M = 4.3 vs. CG 3.5; competence scores M = 4.2
Conversational interaction and social presence	Bashayer chatbot Al-Abdullatif et al. (2023); WeChat apps Zou et al. (2023); Kohnke and Moorhouse (2025)	Relatedness	Reduced isolation; qualitative reports of “human-machine connection”

### 4.2 Moderating factors: gender and academic level

From the perspective of Positive Psychology—which emphasizes that positive outcomes depend on the fit between environmental supports and individual differences—the affective influence of GenAI is non-uniform. Two learner characteristics, gender and academic level, significantly moderate the satisfaction of SDT needs. These characteristics change the degree to which GenAI features successfully fulfill (or fail to fulfill) learners' basic psychological needs for autonomy, competence, and relatedness. For instance, the same personalized feedback may strengthen competence for a novice but become redundant for an expert (academic level moderation); similarly, an AI chatbot may enhance competence satisfaction more strongly for male students than for female students due to differences in technology self-efficacy (gender moderation). These moderating effects require theoretically grounded explanation, which we provide following sections.

#### 4.2.1 Gender disparity in affective response and self-efficacy

Evidence from four reports regarding gender differences in AI perception is nuanced and appears to be moderated by the disciplinary context. In technical and engineering fields, two reports indicate a significant gap: Puzon et al. (2025) found that male engineering students outscored females in the affective component (*p* = 0.029) and AI self-efficacy (*p* = 0.002) (Puzon et al., 2025), while Sajawal and Kittur (2024a) reported that males had significantly higher confidence in using ChatGPT as a learning tool (*p* = 0.043) (Sajawal and Kittur, 2024a). These findings align with broader trends in STEM where pre-existing technological confidence dictates affective responses (Soysal et al., 2022). Conversely, this gap is less evident in general language learning settings. For instance, (Alfaleh et al. 2025) observed no statistically significant gender disparities in AI utility or motivation among general EFL learners (Alfaleh et al., 2025). Furthermore, Shu and Xu (2022) found that in the Chinese university context, female students actually demonstrated significantly higher overall AI adaptability than males (M = 3.54 vs. 3.39) (Puzon et al., 2025). Consequently, while a male advantage in AI self-efficacy is prominent in vocational technical contexts, it cannot be generalized to the broader EFL learner population.

This pattern directly reflects differential satisfaction of the competence need within SDT. Specifically, male students in technical disciplines appear to experience stronger “competence feedback” from AI interactions—they perceive the technology as a tool that validates and enhances their capability. Conversely, female students' lower affective scores and AI self-efficacy likely stem from insufficiently supported technological confidence, rather than any inherent deficit in ability. In other words, the efficiency of competence-need fulfillment is moderated by socio-psychological factors, not by the technology itself. This disparity can be attributed to pre-existing technological self-efficacy shaped by disciplinary contexts. In engineering and other STEM fields, female students may face unmet psychological needs or insufficient support structures, which undermine their emotional engagement and perceived control over AI technologies. Conversely, higher technological self-efficacy among males fosters a sense of capability, leading to more positive attitudes and engagement.

To prevent GenAI from exacerbating existing gender inequalities, educational interventions must prioritize inclusive design principles. Strategies should explicitly target technological confidence among underrepresented groups, particularly in vocational and STEM fields, ensuring equitable access to AI's psychological benefits. Curricula should focus on enhancing AI competencies for all students while actively bridging gender gaps through targeted support and mentorship.

#### 4.2.2 Academic level variation: cognitive load and the expertise reversal effect

The perception and utilization of AI tools vary markedly across academic levels, challenging the feasibility of a universal approach. Undergraduate and first-year students report significantly higher positive perceptions of AI's motivational utility compared to Master's students (*p* = 0.001) (Sajawal and Kittur, 2024a). Cognitive engagement generally increases with academic progression, peaking in the final undergraduate year before declining among advanced learners.

This disparity can be explained by a vague “relevance gap”; also it finds precise theoretical grounding in Cognitive Load Theory and the Expertise Reversal Effect. For novice learners (undergraduates), GenAI serves as an external scaffold that reduces extrinsic cognitive load. By automatically handling mechanical tasks such as grammar correction, vocabulary retrieval, and basic feedback, AI frees limited mental resources, allowing novices to allocate attention to meaning construction and affective regulation (e.g., academic emotion regulation, AER). This reduction in unnecessary load enhances competence satisfaction and psychological well-being, explaining why early-stage learners report high motivational utility. For advanced learners (Master's students), however, the same AI-generated simplifications become counterproductive due to the Expertise Reversal Effect. When learners have already internalized basic linguistic schemas, generic feedback (e.g., simple translations, elementary grammar hints) becomes redundant—it no longer reduces cognitive load but instead imposes extraneous cognitive load. Advanced learners must filter out irrelevant or oversimplified information, adding unnecessary processing costs. Consequently, perceived motivational utility declines sharply.

From the perspective of competence satisfaction, the low-level scaffolds designed for novices (such as basic grammar correction) fail to provide advanced learners with the experience of mastering high-level challenges. According to the expertise reversal effect, when the support does not match the learner's proficiency, it prevents the learner from obtaining the sense of competence that comes from solving difficult, authentic academic tasks. From the perspective of autonomy satisfaction, a similar divergence emerges. Undergraduates achieve a sense of autonomy through completing structured, foundational exercises independently. Master's students, in contrast, require more challenging, exploratory, or research-oriented tasks to experience higher-order autonomy. When AI tools remain at a basic, prescriptive level, this “relevance gap” directly undermines the autonomy satisfaction of advanced learners. Therefore, Master's students require sophisticated support for complex academic discourse, critical analysis, and discipline-specific research writing—not basic auxiliary tools.

To address this, AI applications must be tailored to the cognitive and affective needs of different learner cohorts. For novices, AI should offer structured, competence-building guidance with clear scaffolding. For advanced learners, AI should facilitate open-ended exploration and critical analysis—for example, by replacing generic English texts with discipline-specific journal articles for AI-assisted critical reading, or by allowing AI to be customized or turned off when redundant. Only through such expertise-sensitive design can GenAI remain relevant and motivating across all educational levels.

### 4.3 The double-edged sword: motivation enhancement vs. anxiety induction

The synthesis of the 29 included studies reveals a complex dichotomy: while GenAI consistently serves as a catalyst for motivation, its impact on anxiety remains highly contingent on the human-AI interaction paradigm.

- Theoretical Synthesis of Motivation: The positive impact on motivation is robustly supported across diverse contexts. Quantitatively, the significant gain in intrinsic motivation reported by Dandu et al. (2024) (M = 4.3 vs. 3.5) and the enhancement of Psychological Capital (PsyCap) by Dou and Sun (2025) indicate that GenAI satisfies the SDT-defined need for competence through immediate, formative feedback loops (Dandu et al., 2024; Dou and Sun, 2025). Furthermore, the high demand for personalized paths (79.1%) and the resulting improvement in self-regulated learning (η^2^ = 0.38) suggest that AI fosters autonomy by shifting the locus of control from the teacher to the learner (Wei, 2023). However, a critical examination of Yang et al. (2024) reveals an “engagement paradox”: while learners frequently adopt local linguistic corrections (49%), they often ignore higher-order suggestions (15%) (Yang et al., 2024). This suggests that AI can sometimes promote “mechanical participation” rather than the deep cognitive engagement required for long-term flourishing.
- Resolving the Anxiety Paradox: The evidence concerning anxiety presents a theoretical conflict. For the majority, tools like ASR (Xiao, 2025) mitigate the “fear of negative evaluation” by providing a psychologically safe, private environment (Xiao, 2025). However, the case of Shazly (2021), where anxiety scores rose from 15.3 to 16.8, indicates the presence of Technostress (Shazly, 2021). This divergence can be explained through Cognitive Load Theory and Cognitive Appraisal: when AI interfaces are inflexible or feedback is perceived as an “overseer's judgment” rather than a “supportive scaffold,” learners undergo a negative appraisal, viewing the technology as a threat to their ego.
- Pedagogical Implications for Design: To maximize the positive psychology of AI, design must transition from academic utility to affective safety. Instead of framing AI as an assessment tool, it should be integrated as a low-stakes “virtual peer.” This requires moving beyond mere “correctness” to incorporate emotional intelligence support (demanded by 27.0% of students) (Kohnke and Moorhouse, 2025) and socially situated interaction, ensuring that AI satisfies the need for relatedness—the most frequently neglected pillar of SDT in current AIED design.

### 4.4 Limitations

Despite the growing empirical evidence, several “unknowns” persist and require targeted investigation.

#### 4.4.1 Longitudinal stability of affective gains

It remains unknown whether observed motivational boosts and anxiety reductions represent permanent psychological shifts or temporary “novelty effects.” Longitudinal studies spanning entire degree cycles (e.g., 3–4 years) are needed to assess the stability of Psychological Capital (PsyCap), the internalization of emotion regulation skills, and the durability of AI-induced well-being gain.

#### 4.4.2 AI Emotional intelligence and naturalness

Current GenAI tools lack deep cultural and pragmatic nuance. Moreover, the impact of voice naturalness, prosody, and conversational timing on reducing listening anxiety and enhancing relatedness remains underexplored. Research should investigate emotionally intelligent AI that can detect and respond to behavioral cues of frustration, boredom, or confusion in real time, adapting its tone and support accordingly.

#### 4.4.3 Stakeholder ecosystems: teachers and policymakers

Most research focuses exclusively on end-users (students). Our participant distribution reveals a prominent absence of teachers, instructional designers, administrators, and policymakers, whose beliefs and actions shape the psychological environment for AI use. Future research must adopt an ecological perspective, examining how teacher training, curriculum integration, and administrative policies mediate or moderate the affective impact of GenAI on learners.

#### 4.4.4 Geographical and methodological limitations

Geographical diversification: We state that 27 of the 29 included studies were conducted in Asian and Middle Eastern contexts (China, Saudi Arabia, India, Ethiopia, Philippines, Turkey, Indonesia). We discuss the implications for generalizability, noting that cultural factors (e.g., collectivist educational norms, high-stakes English proficiency pressure) and technological infrastructure may moderate the observed effects. We explicitly call for future research in Western and other under-represented regions (e.g., Latin America, sub-Saharan Africa outside Ethiopia).

Language and publication biases: Our English-only inclusion criterion led to the exclusion of 162 non-English reports, potentially omitting relevant studies published in other languages (e.g., Chinese, Spanish, Arabic, French). Additionally, our search was limited to peer-reviewed articles and conference papers indexed in four databases (Web of Science, Scopus, ERIC, PsycINFO) and accessible via institutional subscriptions; we did not search gray literature or preprints. Consequently, studies with null or negative findings may be underrepresented, which could inflate the apparent positive effects of GenAI on motivation, anxiety reduction, and well-being. Furthermore, the 136 reports excluded because full texts were unavailable (despite inter-library loan attempts) may have disproportionately affected research from lower-income institutions or regions with limited journal access, reinforcing the geographical imbalance noted above. Therefore, our findings are most directly applicable to Asian and Middle Eastern EFL higher education contexts, and generalization to other regions should be made cautiously.

#### 4.4.5 Ethical limitations

Several ethical concerns remain underexplored in the current literature. First, overdependence on AI: Learners may become overly reliant on GenAI for language production, potentially undermining the development of independent linguistic reasoning and problem-solving skills. This overdependence could paradoxically reduce long-term competence satisfaction (a core SDT need) by limiting authentic mastery experiences. Second, hallucinated feedback: GenAI tools sometimes generate plausible but factually incorrect or pedagogically inappropriate responses (“hallucinations”). Such errors may mislead learners, create confusion, or erode trust in AI systems, potentially increasing anxiety rather than reducing it. Third, academic integrity: The low ethical acceptance of using ChatGPT in high-stakes exams (mean 2.27/5; Sajawal and Kittur, 2024) reflects a pervasive “integrity-related stress”. Without clear institutional guidelines, learners face uncertainty about what constitutes legitimate AI assistance vs. plagiarism, which may trigger anxiety and avoidance behaviors. Fourth, reduction of teacher agency: Over-reliance on GenAI could marginalize the role of teachers, reducing their professional autonomy and opportunities for meaningful pedagogical interaction. When AI systems replace rather than augment teacher feedback, the valuable human elements of empathy, contextual understanding, and motivational mentoring may be lost. Fifth, emotional dependency on AI systems: Conversational AI that provides consistent, non-judgmental support may lead learners to form emotional attachments, potentially reducing their willingness to engage in real-world social interactions or seek help from human peers and instructors. This dependency could have unforeseen consequences for social-emotional development. Future research must systematically investigate these ethical dimensions, and educational policies should proactively address them through transparent guidelines, teacher training, and AI literacy programmes.

## 5 Conclusion

This systematic review provides a theoretically grounded synthesis of how Generative AI influences EFL learners' motivation, anxiety, and well-being in higher education. Its unique contribution lies not in simply confirming positive effects, but in demonstrating that GenAI's psychological impact is conditional on design, context, and learner characteristics—a finding that challenges both over-optimistic claims and outright dismissal. By mapping specific AI features to SDT needs (autonomy, competence, relatedness) and identifying gender and academic level as key moderators, we offer a contingent theoretical framework that explains when and for whom GenAI supports or undermines learner psychology.

Practical implications are threefold. For educators, GenAI should be deployed as a low-stakes, formative scaffold rather than an assessment overseer, with explicit attention to reducing technostress. For instructional designers, AI tools must incorporate emotionally intelligent features (e.g., detecting frustration, adapting tone) and inclusive design that proactively builds AI self-efficacy among female students. For policymakers, institutional guidelines should clarify acceptable AI use to reduce integrity-related stress, while ensuring equitable access across gender and academic levels.

Future research should prioritize four under-researched themes: (1) longitudinal studies tracking whether affective gains persist beyond novelty effects; (2) emotionally intelligent AI capable of real-time responsiveness to learners' affective states; (3) inclusive AI design that systematically addresses gender and disciplinary disparities in AI self-efficacy; and (4) culturally responsive AI pedagogy that validates findings across Western and under-represented educational contexts. Addressing these unknowns will determine whether GenAI fulfills its promise as a genuinely human-centered educational technology.

## Data Availability

The original contributions presented in the study are included in the article/Supplementary material, further inquiries can be directed to the corresponding author.
