# Supplementary material for: The positive psychology of AI: a systematic review of generative AI's impact on EFL learners' motivation, anxiety, and well-being in higher education
Source: Front Psychol. 2026 Jul 2;17:1762411. doi: 10.3389/fpsyg.2026.1762411 (PMC13375186; doi:10.3389/fpsyg.2026.1762411)
Supplement: Supplementary file 1 [file Supplementary_file_1.docx]

**Appendix A: Quality Appraisal of the 29 Included Studies (MMAT 2018)**

| **No.** | **Study** | **Design** | **Sampling** | **Measurement** | **Confounding** | **Data completeness** | **Analysis** | **Overall bias** |
| --- | --- | --- | --- | --- | --- | --- | --- | --- |
| 1 | Kohnke & Moorhouse (2025) | Qualitative | Yes | Yes | N/A | Yes | Yes | Low |
| 2 | Shu & Xu (2022) | Survey | Yes | Yes | Yes | Yes | Yes | Low |
| 3 | Khasawneh et al. (2025) | Quasi‑exp | Yes | Yes | No | Yes | Yes | Moderate |
| 4 | Alfaleh et al. (2025) | Survey | Yes | Yes | Yes | Yes | Yes | Low |
| 5 | Huang et al. (2023) | Quasi‑exp | Yes | Yes | No | Yes | Yes | Moderate |
| 6 | Dandu et al. (2024) | RCT | Yes | Yes | Yes | Yes | Yes | Low |
| 7 | Puzon et al. (2025) | Survey | Yes | Yes | Yes | Yes | Yes | Low |
| 8 | El Shazly (2021) | Case study | No | Yes | No | Yes | Yes | Moderate |
| 9 | Batdi (2024) | Meta‑analysis | Yes | Yes | Yes | Yes | Yes | Low |
| 10 | Shi et al. (2025) | Survey | Yes | Yes | Yes | Yes | Yes | Low |
| 11 | Al‑Abdullatif et al. (2023) | Quasi‑exp | Yes | Yes | No | Yes | Yes | Moderate |
| 12 | Song & Song (2023) | Mixed‑methods | Yes | Yes | No | Yes | Yes | Moderate |
| 13 | Yang et al. (2024) | Case study | No | Yes | N/A | Yes | Yes | Moderate |
| 14 | Wang & Xue (2024) | Intervention | Unclear | Yes | No | No | Yes | High |
| 15 | Wei (2023) | Quasi‑exp | Unclear | Yes | No | Yes | Yes | Moderate |
| 16 | Zou et al. (2023) | Quasi‑exp | Yes | Yes | No | Yes | Yes | Moderate |
| 17 | Zou et al. (2023) | Mixed‑methods | Yes | Yes | N/A | Yes | Yes | Moderate |
| 18 | Zou et al. (2023) | Mixed‑methods | Yes | Yes | N/A | Yes | Yes | Moderate |
| 19 | Fathi & Rahimi (2024) | Qualitative | Yes | Yes | N/A | Yes | Yes | Low |
| 20 | Yin et al. (2024) | Mixed‑methods | Unclear | Yes | N/A | Yes | Yes | Moderate |
| 21 | Yuan & Liu (2025) | Intervention | Unclear | Yes | No | No | Yes | High |
| 22 | Weng & Chiu (2023) | Systematic review | N/A | N/A | N/A | N/A | N/A | Not rated* |
| 23 | Xiao (2025) | RCT | Yes | Yes | Yes | Unclear | Yes | Moderate |
| 24 | Wang & Wang (2024) | Theoretical | N/A | N/A | N/A | N/A | N/A | Not rated* |
| 25 | Zawacki‑Richter et al. (2019) | Systematic review | N/A | N/A | N/A | N/A | N/A | Not rated* |
| 26 | Prégent et al. (2025) | Scoping review | N/A | N/A | N/A | N/A | N/A | Not rated* |
| 27 | Dou & Sun (2025) | Quasi‑exp | Unclear | Yes | No | Yes | Yes | Moderate |
| 28 | Yilmaz & Karaoglan Yilmaz (2023) | RCT | Yes | Yes | Yes | Yes | Yes | Low |
| 29 | Hawanti & Zubaydulloevna (2023) | RCT | Yes | Yes | Yes | Yes | Yes | Low |

*Systematic reviews and theoretical papers (Nov. 22, 24, 25, 26) were not rated for bias as they provided contextual or secondary evidence; they were not used as primary sources for causal claims. Inter-rater agreement:κ = 0.84^#^.

^#^Inter‑rater agreement for the overall risk‑of‑bias classification (low / moderate/ high) of the remaining 25 empirical studies was assessed using Cohen’s Kappa (κ). The observed agreement rate (*Po*) was 92% (23 out of 25 studies rated identically by both reviewers). The expected chanceagreement (*Pe*) , calculated from the marginal distributions of the ratings, was 0.47. Applying the $\kappa=(Po-Pe)/(1-Pe)$ yields κ=(0.92−0.47)/(1−0.47)=0.45/0.53≈0.84. According to Landis & Koch (1977), a κ value of 0.84 indicates “almost perfect” agreement beyond chance.

​
